# Gut microbiome and human health: Exploring how the probiotic genus *Lactobacillus* modulate immune responses

**DOI:** 10.3389/fphar.2022.1042189

**Published:** 2022-10-24

**Authors:** Sonakshi Rastogi, Aditi Singh

**Affiliations:** Amity Institute of Biotechnology, Amity University Uttar Pradesh, Lucknow, India

**Keywords:** lactobacillus, inflammation, gut microbiome, probiotics, gut-lung axis, gutbrain axis, gut-heart axis, gut-bone axis

## Abstract

The highest density of microbes resides in human gastrointestinal tract, known as “Gut microbiome”. Of note, the members of the genus *Lactobacillus* that belong to phyla Firmicutes are the most important probiotic bacteria of the gut microbiome. These gut-residing *Lactobacillus* species not only communicate with each other but also with the gut epithelial lining to balance the gut barrier integrity, mucosal barrier defence and ameliorate the host immune responses. The human body suffers from several inflammatory diseases affecting the gut, lungs, heart, bone or neural tissues. Mounting evidence supports the significant role of *Lactobacillus* spp. and their components (such as metabolites, peptidoglycans, and/or surface proteins) in modulatingimmune responses, primarily through exchange of immunological signals between gastrointestinal tract and distant organs. This bidirectional crosstalk which is mediated by *Lactobacillus* spp. promotes anti-inflammatory response, thereby supporting the improvement of symptoms pertaining to asthma, chronic obstructive pulmonary disease (COPD), neuroinflammatory diseases (such as multiple sclerosis, alzheimer’s disease, parkinson’s disease), cardiovascular diseases, inflammatory bowel disease (IBD) and chronic infections in patients. The metabolic disorders, obesity and diabetes are characterized by a low-grade inflammation. Genus *Lactobacillus* alleviates metabolic disorders by regulating the oxidative stress response and inflammatory pathways. Osteoporosis is also associated with bone inflammation and resorption. The *Lactobacillus* spp. and their metabolites act as powerful immune cell controllers and exhibit a regulatory role in bone resorption and formation, supporting bone health. Thus, this review demonstrated the mechanisms and summarized the evidence of the benefit of *Lactobacillus* spp. in alleviating inflammatory diseases pertaining to different organs from animal and clinical trials. The present narrative review explores in detail the complex interactions between the gut-dwelling *Lactobacillus* spp. and the immune components in distant organs to promote host’s health.

## 1 Introduction

The human body is inhabited by trillions of dynamic and diverse microbial communities that potentially regulate the physiology of the host, popularly known as the “Microbiome” (Malard et al., 2021). The human microbiome performs imperative biological functions such as immune system homeostasis, regulation of host metabolism, prevention of pathogens invasion and improvement of the epithelial barrier function (Malard et al., 2021). The highest density of microbes resides in human gastrointestinal tract, known as the “Gut microbiome” (Dwivedi et al., 2021. The predominant bacterial phyla in the gastrointestinal tract are Firmicutes and Bacteroidetes, followed by Actinobacteria and Proteobacteria (Rinninella et al., 2019). Of note, the members of the genus *Lactobacillus* that belong to phyla Firmicutes are the most important probiotic bacteria of the gut microbiome. These gut-residing *Lactobacillus* species not only communicate with each other but also with the gut epithelial lining to balance the gut barrier integrity, mucosal barrier defence and ameliorate the host immune responses (Martín et al., 2019). In addition, *Lactobacillus* species exhibit microbial roles by competitively excluding opportunistic pathogens from inhabiting functional niches in the gut, restraining attachment of pathogens on epithelium as well as directly killing pathogens by producing lactic acid, acetic acid, propionic acid, bacteriocins and reactive oxygen species (ROS) (Dempsey and Corr, 2022). Besides microbial roles, commensal *Lactobacillus* species regulate both adaptive and innate immune responses by inducing T cells, Natural Killer (NK) cells, macrophages differentiation, cytokine-production and stimulating toll-like receptors (TLRs). They also manifest immunomodulatory effect by increasing immunoglobulin-A (IgA) producing B cells expression in Peyer’s patches in the lamina propria, where they block pathogens adhesion to the intestinal epithelium (Cristofori et al., 2021). The human body suffers from several inflammatory diseases affecting the gut, lungs or neural tissues. Mounting evidence supports the significant role of *Lactobacillus* species in suppressing inflammatory responses by downregulating the expression of T-helper17 (Th17) inflammatory cells and their signature cytokines IL-17F, and tumor necrosis factor-alpha (TNF-α). Numerous works of literature is available that highlights the beneficial role of these commensals in ameliorating symptoms of cardiovascular-related diseases (CVD), osteoporosis, metabolic diseases such as diabetes and obesity (Zaiss et al., 2019; Archer et al., 2021; Companys et al., 2021). The genus is also reported to regulate cholesterol metabolism as well as gut-derived metabolites production such as trimethylamine-N-oxide (TMAO), short chain fatty acids (SCFAs), lipopolysaccharides (LPS), and bile acids (BA). Osteoporosis is associated with bone inflammation and resorption (Britton et al., 2014). The *Lactobacillus* spp. and their metabolites also act as powerful immune cell controllers and exhibit a regulatory role in bone resorption and formation, supporting bone health. (Zaiss et al., 2019).

In view of these considerations, the significant role of *Lactobacillus* spp. in the alleviation and treatment of human diseases presents attractive therapeutic potential. Thus, the aim of this review is to analyze the current research works implicating the potent use of these microbes and their components in mediating immune responses that affect host health. PubMed was used to search for all of the studies published over the last 15 years using the key words “*lactobacillus*” or “Gut microbiota” and “inflammation”. More than 550 articles were found, and only those published in English and providing data on aspects related to human diseases were included in the evaluation.

## 2 Genus *Lactobacillus* as a member of the gut microbiome

Nowadays, the gut microbiome is a prominent area of scientific research as it holds imperative role in human health and pathology. Of note, the members of genus *Lactobacillus* are the most important probiotic bacteria. *Lactobacillus* spp. act by regulation of luminal pH, enhancement of barrier function by increasing mucus production, secretion of antimicrobial peptides, and by changing the gut microbial composition (Dempsey and Corr, 2022). Their cogent functional attributes include improving digestion, maintenance of gastrointestinal-barrier integrity, competition to opportunistic pathogens, neuromodulation, participation in maturation of the immune system in early life and preservation of immune homeostasis during entire life, production of metabolites, vitamins and other components (Dempsey and Corr, 2022).

Detailed taxonomic profiling reveals that the genus *Lactobacillus* belongs to phylum Firmicutes, class Bacilli, order Lactobacillales and family Lactobacillaceae. They are Gram-positive, catalase-negative, non-spore forming, obligate saccharolytic rods or coccobacilli with low GC (guanine and cytosine) content of the genome (Zheng et al., 2020). Members of the genus *Lactobacillus* are well-adapted to the hostile environment persisting in oral, gastrointestinal and vaginal tract. In healthy adult’s feces, the concentration of different lactobacilli species accounts for upto 10^5^–10^8^ CFU/g. Among human gut-dwelling microbial species*, Lactobacillus crispatus, Lactobacillus gasseri, Lactobacillus ultunensis, Ligilactobacillus ruminis, Limosilactobacillus reuteri, Lactobacillus kalixensis, Lactocaseibacillus casei, Limosilactobacillus gastricus, Limosilactobacillus antri, Lactobacillus rhamnosus, Ligilactobacillus salivarius, Limosilactobacillus fermentum, etc.* are found to be permanent and form essential part (Zheng et al., 2020). Probiotic supplementation with lactobacilli species assists in altering the gut microbiota composition, thereby reducing dysbiosis and maintaining the microbial balance with a greater abundance of useful bacteria. A probiotic formulation comprising of *L. rhamnosus* GG, *L. acidophilus*, *L. plantarum*, *L. paracasei*, and *L. delbrueckii* when given orally tends to stimulate the other gut microbial species such as Prevotella and Oscillibacter that exhibited anti-inflammatory activity in rats (Zeng et al., 2021).

## 3 Genus *Lactobacillus* and human health

As probiotics, lactobacilli play significant roles through various GM-derived metabolites andpossess several health ameliorating attributes common among them are alleviation of chronic diseases, immune system stimulation, pathogen protection, and nutritional physiology.

### 3.1 Gastrointestinal barrier integrity

When the gut barrier becomes dysfunctional, it leads to several inflammatory conditions, such as irritable bowel syndrome (IBS), inflammatory bowel disease (IBD) and obesity. Gut-dwelling *Lactobacillus* spp. were found to restore gastrointestinal barrier function directly or indirectlyin mice inflammation models (Martín et al., 2019). Indirect mechanisms through which they interact with the immune system involve modulating gut microbiota, regulating intestinal epithelial barrier integrity, viscoelastic mucin layer properties, antagonistic peptides/factors production and competitive exclusion of pathogens (Cristofori et al., 2021).

#### 3.1.1 Gut epithelial barrier

The gut epithelial barrier separates the internal intestinal milieu from the luminal environment, thereby ascertaining the permeability of nutrients and other molecules as well as exerting a protective role by arresting the entry of microbes and toxic compounds. Studies have reported that commensal Lactobacilli spp. maintain gut barrier integrity which depends upon the multi-protein complexes such as tight junctions, gap junctions, adherens and desmosomes (Kocot et al., 2022). These complexes are primarily made up of transmembrane proteins namely, claudin, occludin, and junctional adhesion molecules which interact with adjacent cells *via* zonula occludens (ZO) and actin fibers. In case of chronic inflammatory diseases or enteric infections, the intestinal barrier integrity is disrupted. Studies have reported increased expression of ZO-1, occludin and claudin proteins by *L. rhamnosus* CNCM-I in enterohemorrhagic *E. coli* O 157:H7 infected Caco-2 cell lines (Laval et al., 2015). Similarly, *L. casei* DN-114 001 up-regulates ZO-1 protein expression *via* activation of Toll-like receptor (TLR)-2 in Caco-2 cells. The TLR-2 binding leads to Protein kinase C (PKC) activation which causes tight junction proteins translocation, thereby enhancing gut barrier function (Kocot et al., 2022).

#### 3.1.2 Mucus production

Gut epithelium is covered by high molecular weight glycoproteins, mainly mucins which are produced by goblet cells (Wuet al., 2020). The viscoelastic mucin layer provides protection against digestive enzymes, supports food passage and prevents translocation of microbes across lamina propria, thus imparting gut homeostasis (Wu et al., 2020). Studies have reported certain *Lactobacillus* strains to regulate mucin gene expression thereby changing the mucus layer attributes and indirectly affecting the gut immune responses. Of note, *L. rhamnosus* CNCM I-3690 regulated Muc2 and Muc3 gene expression in mucus-producing goblet cells in mice inflammation model, thus preventing gut barrier dysfunction and inflammation (Martín et al., 2019).

#### 3.1.3 Anti-microbial peptides/factors

The vital function of lactobacilli strains lies in their ability to prevent pathogenic growth by synergistically inhibiting enteropathogens and stimulating the host immune system. The lactobacilli that showed *in vitro* antagonistic activity against periodontal and enteric pathogens are *L. oris*, *L. paracasei*, *L. crispatus*, *L. gasseri*, *L. salivarius*, *L. plantarum*, *L. delbrueckii*, *L. rhamnosus*, *L. acidophilus*, and *L. fermentum*, thereby improving gut homeostasis (NĘdzi-GÓra et al., 2020; Dempsey and Corr, 2022). Among the known factors that contribute to the antimicrobial ability of *Lactobacillus* spp. is their tendency to produce a wide range of metabolites such as organic acids, hydrogen peroxide, nitric oxide, SCFAs and bacteriocin that impede the growth of pathogens (Sulijaya et al., 2020). Organic acids, particularly lactic acid is a potent inhibiting factor as it creates acidic cytoplasmic pH as well as permeabilize the outer membrane of Gram-negative microorganisms (Sulijaya et al., 2020). For example, *L. acidophilus* 4,356 inhibited the growth of *H. pylori* by abundantly producing lactic acid (Modiri et al., 2021). Nitric oxide (NO) is another inhibitory microbial metabolite. Probiotic lactobacilli can effectively elevate NO synthesis or can stimulate host macrophages for NO production. For example, *L. fermentum* LF1 produces NO *via* the NO synthase pathway *via* oxidation of l-arginine to l-citrulline (Dempsey and Corr, 2022). Some may even ribosomally synthesize short peptides known as bacteriocins which possess broad-spectrum inhibitory activity against foodborne pathogens and spoilage bacteria. For example, *L. plantarum* LPL-1 produced 4,347 Da plantaricin LPL-1 (Wang et al., 2018) while curvacin A is from *L.* *curvatus* (Ahsan et al., 2022). In the genome of several other lactobacilli species, genes encoding for pediocin and plantaricin are found (Ahsan et al., 2022). The hydrogen peroxide producing lactobacilli also induce growth stagnation in pathogens. *L. johnsonii* UBLJ01 genome analysis revealed NADH oxidase, lactate oxidase and pyruvate oxidase genes involved in H_2_O_2_ synthesis while *in vitro* test reported their strong antagonistic potential (Ahire et al., 2021). In addition to the synthesis of inhibitory substances, *Lactobacilli spp.* can decrease the toxicity by degradation of microbial toxins, particularly by impeding toxin expression or by binding to the pathogen’s outer membrane. For example, *L. rhamnosus* JB3 reduced the infection of *H. pylori* in the gut by either forming lipid rafts or downregulating the expression of virulent genes (Do et al., 2021).

Recently, literature is available that highlights the ability of *lactobacillus* spp. in detoxifying mycotoxins that are known to cause carcinogenesis and immune-suppression in the host. Of note, *L. coryniformis* BCH-4 and *L. plantarum* MiLAB 393 produced fungicide compounds cyclo (L-Leucyl-L-Prolyl) and 3-phenyllactic acid cyclo (L-Phe-L-Pro)respectively which significantly reduced the viability of aspergillus and *candida* species (Ström et al., 2002; Salman et al., 2022). Additionally, Sunmola et al., 2019 reported the anti-viral activity of *L. plantarum* and *L. amylovorus* AA099 against enteroviruses such as echovirus whose site of primary replication is in the gastrointestinal tract. Similarly, Kawahara et al., 2022 reported the antiviral activity of S-layer proteins from *L. crispatus* KT-11 strains by suppressing the amplification of rotavirus protein 6 (VP6) expression in human intestinal epithelial Caco-2 cells. Also, Mousavi et al., 2018 demonstrated that *L. crispatus* microcolonies formation on the cell surface tends to block the entry of herpes simplex virus-2 particles, thus help in inhibiting the primary infection step. The rotaviruses are among the ones that bring about severe recurrent diarrhea in infants while herpes simplex virus I cause oral herpes in adults (Kawahara et al., 2022). The understanding of the exact mechanisms is still limited but a few of them include the synthesis of sialic acid and bacteriocins, immune stimulation and obstruction in the binding of viruses (Mousavi et al., 2018; Kawahara et al., 2022).

### 3.2 Gastrointestinal inflammatory disorders

Inflammations are physiological responses to tissue injury and/or infections, elicited by pro-inflammatory cytokines produced by monocytes, B cells, T cells, dendritic cells (DC), natural killer (NK) cells, and macrophages. Substantial evidence from colitis-induced murine model studies claims the anti-inflammatory and immunomodulatory action of *Lactobacillus* spp. in gut inflammation (Cristofori et al., 2021). These effects largely depend upon cytokine production and immune cells proliferation. Pro-inflammatory cytokines, such as IL-8 play important role in the recruitment of immune cells during an inflammatory response. Notably, *L. acidophilus* can suppress IL-8 production and enhance Toll-like receptor-2 (TLR-2) expression through the regulation of TLR-2 mediated mitogen activated protein kinase (MAPK) signalling pathways and Nuclear Factor kappa-light chain-activated B cells (NF-кB) in inflammatory epithelial cells of the intestine (Li et al., 2021). Likewise, several strains of *lactobacillus* (such as *L. acidophilus* CCFM137, *L. fermentum* CCFM381, *L. plantarum* CCFM634 and CCFM734) also displayed anti-inflammatory potential by upregulating the expression of TLR-2/TLR-6 heterodimer receptor which act as an inflammatory intracellular signalling network (Ren et al., 2016). Inflammatory bowel disease (IBD) is systemic disorder that significantly perturbs the intestinal epithelial layer of the gastrointestinal tract causing four pathological conditions, Crohn’s disease (CD), ulcerative colitis (UC), microscopic colitis and pouchitis. Compare et al., 2017 reported *L. casei* DG lower pro-inflammatory IL-6, IL-8, TLR-4 and IL-1a and increase IL-10 cytokinelevels in the colonic mucosa of post-infectious IBD subjects. Additionally, *L. casei* and *L. bulgaricus* significantly lowered the pro-inflammatory cytokine TNF-α in colonic mucosal samples from CD patients (De Conno et al., 2022). The Treg cells are another immunological player involved in immune-modulation and tolerance. *L. casei* M2S01 displayed anti-inflammatory action in diseases, such as CD and microscopic colitis, by enhancing Treg cell activation, IL-10 levels and restoring gut microbial flora (Liu Y et al., 2021). Necrotizing enterocolitis (NEC) is another serious gastrointestinal inflammatory condition affecting particularly premature newborns. An *ex-vivo* study carried out on human intestinal cells from the ileus of NEC infants when treated with *L. rhamnosus* HN001 displayed reduced NF-kB inflammatory pathway activation through inhibition of TLR-4 (Good et al., 2014). Furthermore, commensal *lactobacillus* spp. activates mucosal immunity by increasing IgA antibodies, resulting in the immobilization and agglutination of pathogens. The dose-dependent consumption of *lactobacillus* particularly*, L. plantarum*, *L. acidophilus*, *L. casei*, *L. delbrueckii* subsp. *bulgaricus*, *L. rhamnosus* accrued the number of IgA-producing immune cells connected with mucosa lamina propria as well as stimulates the immunoglobin receptors present on epithelial cells of the intestine (Chen et al., 2022).

**FIGURE 1:**
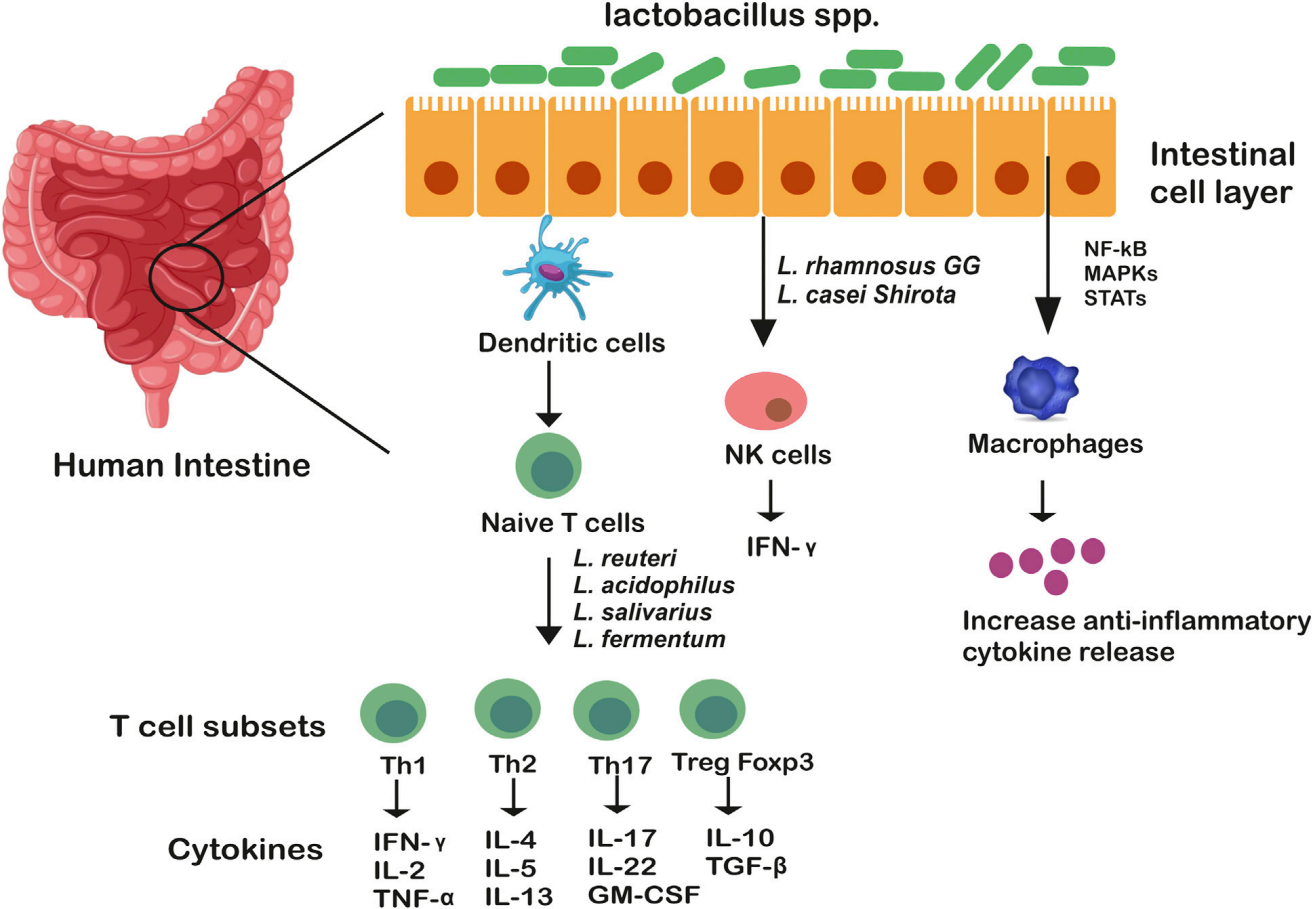
Genus Lactobacillus mediated immune modulation in the gut. Genus *lactobacillus* as whole bacteria or their components communicate through membrane receptors TLR-6 and TLR-2 which are expressed on macrophages and dendritic cells, stimulating T cell subsets differentiation. These T cells (Th1, Th2, Th17, Treg) are considered as the masters of inflammation. *Lactobacillus* spp. also exert their functions by altering intracellular pathways of immune cells (such as macrophages) through MAP kinases which either activate or suppress transcription factors, STAT, NF-kB, increasing anti-inflammatory cytokine release.

Adhering to the host ileal tract, lactobacilli modulate another important aspect, apoptosis and cancer, both of which are associated with mucositis. For example, *L. rhamnosus* GG actively induces antiapoptoticAkt/protein kinase B and inhibits pro-apoptotic factors *via* p36 MAPK pathway while cell wall components of an array of lactobacilli such as lipoteichoic acid tends to stimulate NO synthase, thereby initiating a cascade of events that bring about pathogen-infected cell death (Banna et al., 2017). Important immune events include activation of macrophages through TNF-α cytokine production and NO-mediated upregulation of two surface phagocytosis receptors (FcγRIII and TLR-2). Sun et al., 2020 reported LPS-induced inflammatory response in Caco-2 cells owing to upregulation of anti-inflammatory cytokines genes (IL10, IL4, transforming growth factor-β3 (TGF–b3 and IFN-y)and downregulation of pro-inflammatory cytokines (IL6, IL1B, IL8 and TNF-α) along with higher expression of TLR-2 and NOD-like receptor genes. Also, genome analysis unfolds the activation of phosphatidylinositol 3-kinase (PI3K)/protein kinase B (Akt) signaling pathway in Caco-2 cells by *L. gasseri* JM1. Another *in vivo* study demonstrated induction of innate and adaptive immune responses by *L. acidophilus* NCFB 1748 and *L. paracasei* DC412 on BALB/c inbred mice and Fisher-344 inbred rats through polymorphonuclear (PMN) cell recruitment, TNF-α secretion and phagocytosis (Azad et al., 2018). Lactobacilli encode for certain motifs within their genome, such as unmethylated CpG motifs which are recognized by TLRs that are expressed particularly in B cells and macrophages (Xiao et al., 2022). The role of *Lactobacillus* spp. in modulating immune responses is illustrated in Figure 1.

In recent research, studies involving immunomodulating role of *lactobacillus* spp. in pain management and treating allergic symptoms have gained momentum. To date, few researchers have demonstrated the prowess of *Lactobacillus* spp. in relieving inflammatory pain by regulating pro-inflammatory cytokines such as TNF-α, IL-6 and IL-1β as well as expression of COX II in the host intestinal epithelium (Santoni et al., 2021). Although the exact pathway and mechanisms involved in inflammatory pain relief are yet not investigated in detail. *L. reuteri* DSM17938 displayed antinociceptive activity causing relief in children with functional abdominal pain (Jadrešin et al., 2020). The probable mechanism involves the activation of submucosal immune cells that trigger sensitivity in nerve terminals and/or tight junctions. Another randomized, placebo-controlled, double-blind, multi-centric study by Martoni et al., 2020, reported improvement in abdominal pain and IBS symptoms in adult subjects after the administration of *B. lactis* UABla-12and *L. acidophilus* DDS-1 for over 6 weeks. Maixentet al., 2020 also reported the efficacy of the cocktail of two *L. acidophilus* strains in relieving pain in IBD patients.

### 3.3 Respiratory inflammatory disorders (gut-lung axis)

*Lactobacillus* as whole bacteria or their components (such as peptidoglycans, metabolites and surface proteins) have shown to exert an immunomodulating role in treating patients with chronic respiratory disorders. The probable mechanisms by which *Lactobacillus* modulate lung immunity and promote respiratory health is *via* the “Gut-Lung axis” which is bidirectional (Du et al., 2022).

In the intestinal mucosa, pattern recognition receptors (PRRs, such as NLRs or TLRs) present on immune cells recognize *Lactobacillus* species or their components resulting in the activation of innate immune cells which tend to reach pulmonary tissues through lymphatic circulation (Stavropoulou et al., 2021; Du et al., 2022). To elucidate, oral administration of *L. paracasei* CNCM I-1518 caused innate lymphoid group 3 cells (ILC3s) to migrate from the gut to the lungs where ILC3 provides resistance to pneumonia (Gray et al., 2017). Moreover, *L. rhamnosus* GG and *L. murinus* oral supplementation promotes the migration of Treg cell to the lungs, thereby augmenting pulmonary inflammation (Zhang et al., 2018; Han et al., 2021). The Treg cells not only have the potent anti-inflammatory ability but are also found to block Th2 type immune response in the host. In addition to this, *Lactobacillus* interaction with gastric mucosa results in the secretion of cytokines by immune cells, which through circulation reach lung tissues where they alter the immune response. Kollinget al., 2018 reported oral intake of *L. rhamnosus* CRL1505 results in higher TNF-α, IFN-β, IFN-α, and IFN-ɣ cytokines levels in the lungs leading to a significant reduction in respiratory inflammations. Of note, certain *lactobacillus* strains secrete metabolites, particularly short chain fatty acids (SCFAs) such as acetate, propionate, butyrate that can regulate host pulmonary immune responses. SCFAs affect immune response in two ways; one is when unmetabolized SCFAs directly migrate to the lungs through circulation and enhance G protein-coupled receptors (GPCRs) activation or histone deacetylase inhibition (Koh et al., 2016). The other is where SCFAs migrate to bone marrow through circulation where they increase the differentiation of macrophage and dendritic progenitor cells (MDPs) and convert them into Ly6C–monocytes, these in turn reach the lungs and differentiate into anti-inflammatory alternatively activated macrophages (AAMs). These AAMs reduce neutrophiles recruitment and stimulate Treg cells to produce anti-inflammatory cytokines (IL-10, TGF-β), thus lower lung injury and inflammation (Anand and Mande, 2018; Du et al., 2022). Animal studies have reported higher butyrate production leads to an increase in Tregs cells and IL-10 production mediated by GPR109A receptor activation as well as restoration of IL-10 in pulmonary tissues by inhibiting histone deacetylase (Vieira et al., 2019). Spacova et al., 2020 showed that *L. rhamnosus* GR-1 significantly prevented the severity of airway inflammation and hyperactivity by modulating Th2-mediated immune responses as well as shifting gut microbiome composition in the allergic asthma model, supporting the existence of gut-lung axis. Likewise, Li et al., 2019 investigated the efficacy of six *lactobacillus* spp. (*L. fermentum*, *L. salivarius*, *L. rhamnosus*, *L. casei*, *L. reuteri* and *L. gasseri*) on the gut microbiome and airway inflammation of house dust mite (HDM)-treated asthmatic mice models. Among these, strains of *L. reuteri* displayed improved airway inflammation with reduced total HDM-IgG1, IgE and Th2-associated pro-inflammatory cytokines with a shift in gut microbial flora. To summarize, members of *Lactobacillus* spp. or their metabolites have potent anti-inflammatory response, thereby supporting the alleviation of symptoms of asthma, respiratory tract infections and chronic obstructive pulmonary disease (COPD) in patients. The animal and clinical trials associated with *Lactobacillus* spp. in inflammatory disorders of various organ systems are summarized in Table 1.

**TABLE 1:** Animal and clinical studies of different *Lactobacillus* strains in alleviating inflammatory diseases pertaining to different organs.

*Lactobacillus*	Strain	Diseases	Experimental model	Immunological response	Clinical outcomes	References
L. casei	Lbs2	Gastrointestinal inflammatory disorders	TNBS -induced colitis in mice	Increased Treg levels	Lower gastric inflammation	Thakur et al. (2016)
L. plantarum	CCFM634	Gastrointestinal inflammatory disorders	DSS-induced colitis in mice	Stimulated TLR2/TLR6 heterodimer	lower gastric inflammation	Ren et al. (2016)
L. plantarum	CCFM734					
L. fermentum	CCFM381					
L. acidophilus	CCFM137					
L. reuteri	LMG P-2748	Gastrointestinal inflammatory disorders	*C. difficile* induced colitis in mice	Increased IL-10 levels	lower gastric inflammation	Sagheddu et al. (2020)
L. fermentum	CECT5716	Gastrointestinal inflammatory disorders	DSS-induced colitis in mice	Decreased IL-1b, IL-12 and TGF-β levels	lower gastric inflammation	Rodríguez-Nogales et al. (2017)
L. salivarius	CECT5713					
L. rhamnosus	RC007	Gastrointestinal inflammatory disorders	TNBS- induced colitis in mice	Increases IL-10/TNF-α ratio	lower gastric inflammation	Dogi et al. (2016)
L. fermentum	CQPC04	Gastrointestinal inflammatory disorders	DSS-induced colitis in mice	Increases IL-10, decreases TNF-α, IFN-ɣ, IL-1b, IL-6, and IL 12 and Inhibited NF-kBp65, COX-2	Lower inflammatory pain	Zhou et al. (2019)
L. rhamnosus	GG	Respiratory inflammatory disorders	Infant C57BL/6 mice or seven-week-old female BALB/c mice	Increased IL-10 levels	Improvement in survival rate and reduction in lung inflammation	Kumova et al. (2019)
L. plantarum	CIRM653	Respiratory inflammatory disorders	6-8-week-old C57/BL6J mice		The pulmonary inflammation response is reduced	Vareille-Delarbre et al. (2019)
L. casei	CRL 431	Respiratory inflammatory disorders	Adult 8-week-old Swiss albino mice and immune-deficient Swiss albino mice	Lung bacterial load is decreased	Lung inflammation is reduced, accelerated weight recovery	Zelaya et al. (2013)
L. fermentum	CJL-112	Respiratory inflammatory disorders	Female, specific pathogen-free (SPF) BALB/c mice	Significant up-regulation of Th1 cytokine and IgA	Improvement in pulmonary inflammation	Yeo et al. (2014)
L. kunkeei	YB38					
L. murinus	CNCM I-5314	Respiratory inflammatory disorders	Six-eight-week-old female SPF C57BL/6 mice	Increases lung Th17 and RORγt^+^ Tregs cells	Reduction in pulmonary inflammation	Bernard-Raichon et al. (2021)
L. casei	CCFM419	Diabetes	High fat and streptozotocin (HFD/STZ)-induced C57BL/6J mice	Decrease in TNF-α and IL-6, increase GLP-1 and SCFAs levels	Reduced type 2 diabetes, GM modulation	Wang et al. (2017)
L. paracasei	NL41	Diabetes	Eighteen (HFD/STZ)-induced Sprague-Dawley (SD) rats		Decreases insulin resistance, and HbA1c, glucagon, and leptin levels, GM modulation	Zeng et al. (2019)
L. acidophilus	KLDS1.1003	Diabetes	High fat and streptozotocin (HFD/STZ)-induced C57BL/6J mice	Decrease in IL-8, TNF-α and IL-1β in liver and colon. Downregulated expression of glycogen synthase kinase 3β (GSK-3β), fatty 41 acid synthase (FAS) and sterol regulatory element-binding transcription factor 1c 42 (SREBP-1c), up-regulate the expression of protein kinase B (Akt)	Reduced inflammation in colon and liver associated with diabetes, reshape GM	Yan et al. (2019)
L. gasseri	LG2055	Obesity	C57BL/6 mice	Upregulation of pro-inflammatory genes, CCL2 and CCR2. Reduction in hepatic lipogenic genes ACC1, FAS and SREBP1	Decrease in body weight, epididymal fat tissue mass	Miyoshi et al. (2014)
L. brevis	OPK-3	Obesity	Male C57BL/6 mice	Decrease in TNF-α, IL-6, and IL-1β, serum TG levels	Decrease in body weight, epididymal fat tissue mass	Park et al. (2020)
L. fermentum	MTCC:5898	Cardiovascular diseases	Male Wistar rats	Decrease in TNF-α and IL-6	Decrease in coronary artery risk index, atherogenic index, hepatic lipids, lipid peroxidation, serum total cholesterol, LDL, triglycerides	Yadav et al. (2019)
L. brevis	BCRC 12310	Cardiovascular diseases	Six -week-old SH rats	Decrease in the COX-1, COX-2, iNOS, and TNF-α levels	Anti-hypertensive effect by blocking LPS-induced NO production and	Chang et al. (2016)
L. fermentum	ME-3	Cardiovascular diseases	Human subjects	Decrease in IL-6	Decrease in serum total cholesterol, HDL LDL, triglyceride, oxLDL, hsCRP and HB1Ac levels	Kullisaar et al. (2016)
L. paracasei	FZU103	Cardiovascular diseases	HFD-fed BALB/c mice	LXR/inflammatory axis of LPS-stimulated alveolar macrophages, shift in GM	Increase cholesterol metabolism, BAs homeostasis	Lv et al. (2021)
L.rhamnosus	GR-1	Cardiovascular diseases	Eight-week-old ApoE^−/−^ BALB/c mice	Up-regulated NF-κB signaling pathway	Decreased atherosclerotic lesion size, oxidative stress, chronic inflammation	Fang et al. (2019)
L. paracasei	DSM13434	Bone Heath	Six-week-old C57BL/6N female mice	Decrease in TNF-α and IL-1β	Increase in OPG levels, alleviated ovariectomy-induced bone loss and resorption	Ohlsson et al. (2014)
L.reuteri	ATCC 6475	Bone health	Eleven-week old male BALB/c mice	Upregulated Wnt10b signaling, and TNF-α levels	Alleviated glucocorticoid -induced bone loss, shift in GM with reduction in *Clostridium*	Schepper et al. (2019)
L. reuteri	ATCC 6475	Bone health	Fourteen-week C57BL/6 male mice	Decrease in TNF-α levels, Wnt10b signalling suppression	Alleviated type-I diabetes-induced bone loss	Zhang et al. (2015)
L. reuteri	ATCC PTA 6475	Bone health	Twelve- weeks BALB/c mice	Decrease in Osteoclast markers (Trap5 and RANKL), increase in CD4^+^ T cells	Alleviated ovariectomy-induced bone loss	Britton et al., 2014
L. plantarum	C88	Liver injury	Aflatoxin (AFB1) induced 6-week old male ICR mice	Decrease in IL-6, IL-8,IFN- ɣ, TNF-α. Downregulated NF-kB signaling pathway. Decreased levels of Bax and caspase-3, caspase-8 and elevated Bcl-2 levels in liver	Reduced inflammation and apoptosis in liver tissues	Huang et al. (2019)
L. plantarum	Gut-resident	Neuroinflammation	Eight-week adult C57BL/6 mice	Blocked TLR4, IL-1β, IL-6, TNF-α, and MCP1 in brain regions	Mitigating neuroinflammation	Shukla et al. (2022)

### 3.4 Neuroinflammatory and Neurodegenerative disorders (gut-brain axis)

The role of gut-resident *Lactobacillus* species in human brain development and functions has also been reported. The mechanism involves the exchange of neural, hormonal and immunological signals between the gastrointestinal tract and the central nervous system (CNS), primarily known as the“Gut-Brainaxis”. This bidirectional communication is mediated by tryptophan precursors and microbial metabolites such as gamma-aminobutyric acid (GABA), histamine, 5-hydroxytryptamine (5-HT), glutamine, LPS, branched-chain amino acid (BCAAs), bile acids, SCFAs, and catecholamines which regulate potent pathways that are implicated in neuroglial cell function, neurogenesis, myelination, blood–brain barrier function and synaptic pruning (Suganya and Koo, 2020). Of note, human intestinal isolates, lactobacilli were able to produce GABA in the enteric nervous system (ENS). Studies also reported the modulation of the gut microbiota on supplementation with *L. rhamnosus* JB-1 activated the expression of γ-aminobutyric acid (GABA) receptors thereby resulting in marked improvement in cognitive responses in mice *via* the vagus nerve (Breit et al., 2018). Several species of *Lactobacillus* have shown marked effects as neuromodulators and neurotransmitters (such as monoamines, serotonin, and brain-derived neurotrophic factor) (Suganya and Koo, 2020). The microbial metabolite, such as SCFAs produced primarily by lactobacilli in the gut directly affect brain neurological functions through vagal, endocrine, humoral and immune pathways by either entering circulation or crossing the blood-brain barrier (BBB). SCFAs tend to activate Treg cells, endocrine cells and neuronal cells directly in order to increase regulatory cytokines level that maintains brain functions (Silva et al., 2020). LPS induces neurodegenerative and neuroinflammatory disease through TLR stimulation, especially TLR4 in the microglial cells and astrocytes. Studies reported that LPS/TLR4 signaling on microglial cells affects the CNS, particularly by enhancing inflammatory cytokines levels in the gut or CNS of Autism spectrum disorder (ASD) patients. Liu et al., 2019 reported oral supplementation of *L. plantarum* PS128 for 1 month drastically ameliorated ASD-related symptoms as compared with the placebo group in a double-blind, randomized, placebo-controlled study.

**FIGURE 2:**
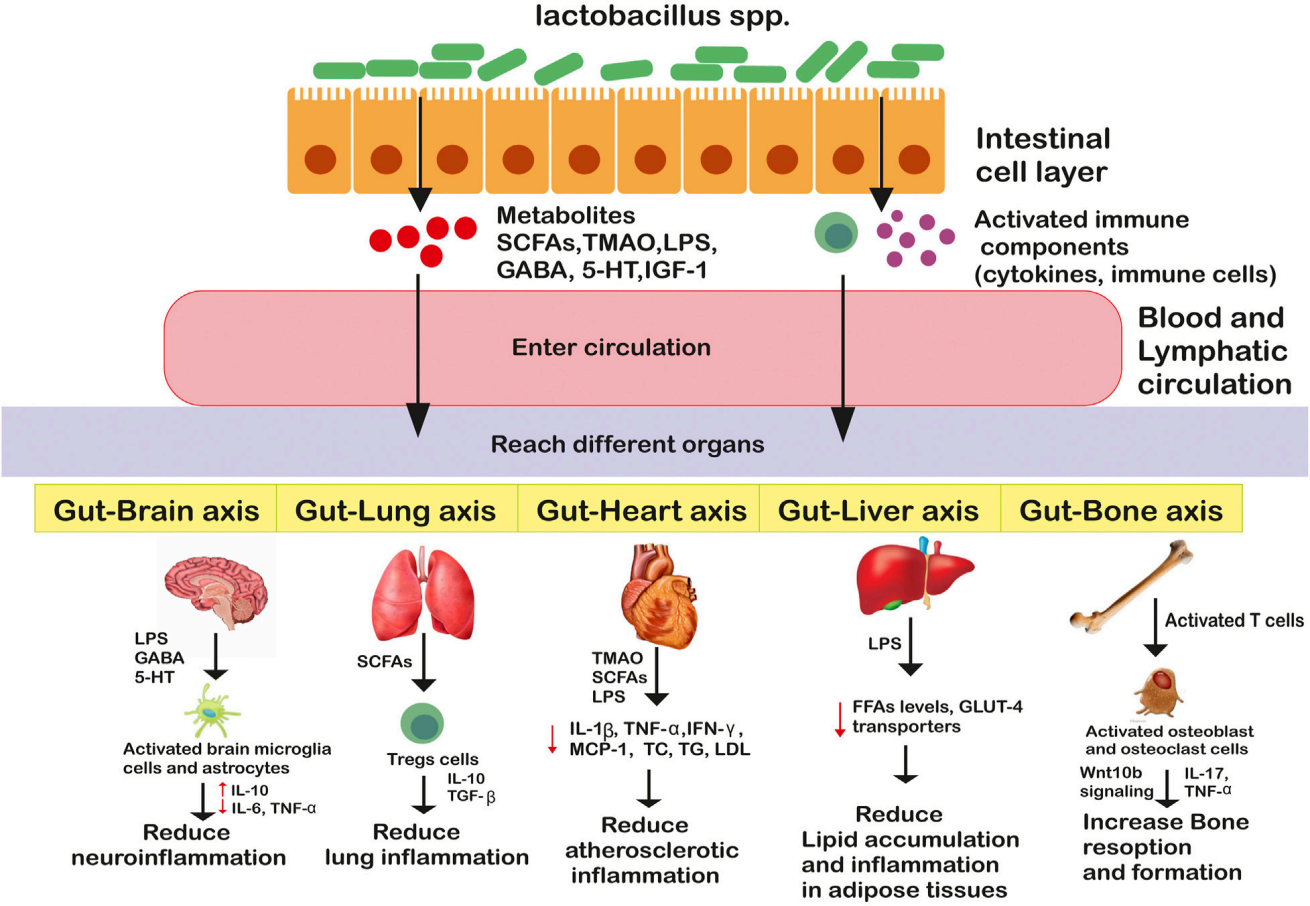
Genus Lactobacillus mediated bidirectional crosstalk affecting host’s health. Genus *lactobacillus* through metabolites and/or activated immune components enter circulation and reach distant organs where they enter different pathways promoting or suppressing inflammatory responses.

Existing evidence supports the significant role of *Lactobacillus* species and their beneficial metabolites in alleviating neuroinflammatory and neurodegenerative disorders in experimental models or clinical settings. Multiple sclerosis (MS) is a neuroinflammatory autoimmune disease that is characterized by myelin sheath degradation and axonal damage (Suganya and Koo, 2020). Kwon et al., 2013 reported that oral administration of *L. casei*, *L. acidophilus,* and *L. reuteni* to experimental mice resulted in delayed MS progression by enhancing Foxp3+ and IL10 + Tregs expression and reducing the pro-inflammatory Th1/Th17 polarization in the peripheral immune system and inflammation site. Another similar research showed suppressed MS symptoms on supplementation with *L. paracasei* DSM 13434 and *L. plantarum* DSM 15312, particularly mediated by IL-10 producing CD4^+^CD25+Tregs on mice (Lavasani et al., 2010). In addition to this, human gut isolates *L. reuteri* NK33, *L. mucosae* NK41, *B. longum* NK46, and B. adolescentis NK98 have been reported to reduce stress-induced anxiety/depression in mice. The probable mechanisms involve modulation of gut microbial composition and inflammatory immune responses by blocking the NF-κB pathway and reducing LPS, corticosterone, IL-6, and TNF-α levels in serum (Jang et al., 2019). Alzheimer’s Disease (AD) is a neurodegenerative disorder wherein considerable decline in memory, activities, cognitive ability and thinking is observed in older adults (Suganya and Koo, 2020). Studies showed intake of *L. acidophilus*, *B. bifidum* and *B. longum* in rats for 12 weeks significantly augmented their spatial learning and memory, long-term potentiation (LTP), paired-pulse facilitation (PPF) ratios, and lipid profiles (Rezaei Asl et al., 2019). Further, Castelli et al., 2020 supported the neuroprotective role of *Lactobacillus* spp. in human neuroblastoma cells, by activating pTrK, P13K/Akt, p-CREB, pERK5 pathways and restoring gut microflora. Parkinson’s disease (PD) is another common neurodegenerative disorder marked by mood deflection, cognitive disturbances, resting tremors, slowness of movement, autonomic dysfunctions, sensory and sleep alternations, involving both the central and peripheral nervous system (Suganya and Koo, 2020). Studies have supported the role of *L. acidophilus* (LA02) and *L. salivarius* (LS01) in reducing pro-inflammatory cytokines (TNF-α, IL-6, IL-17A) and reactive oxygen species (ROS) levels and increasing anti-inflammatory cytokines (IL-4, IL-10) in peripheral blood mononuclear cells (PBMCs) thereby relieving Parkinson’ disease (PD)-associated symptoms (Magistrelli et al., 2019). The intricate crosstalk between gut and distant organs is illustrated in Figure 2.

### 3.5 Cardiovascular diseases (gut-heart axis)

A plethora of literature is available that documented the role of gut-dwelling *Lactobacillus* species in alleviating cardiovascular diseases (CVD) that specifically include diseases of the heart and blood vessels such as congestive heart failure, myocardial infarction, atherosclerosis, coronary artery disease, angina, peripheral vascular disease and aneurysm (Companys et al., 2021). Hypertension and high serum cholesterol are one of the major predisposing factors for CVD. Intestinal *Lactobacillus* colonies exert protective effect on the heart by regulating atheroinflammatory response, cholesterol metabolism, oxidative stress response, and gut-derived functional metabolites production such as TMAO, SCFAs, LPS, and BA (Companys et al., 2021; Papadopoulos et al., 2022). Of note, researchers also support that *lactobacillus* interventions could significantly impart a cardioprotective role by regulating the abundance and diversity of selective gut microbial flora (Zhao et al., 2021). To exemplify, *L. salivarius* Ls-33 supplementation changed gut microbiota composition by escalating the Prevotella-Bacteroides–Porphyromonas group/Firmicutes ratio in obese heart patients (Zhao et al., 2021).

In the human body, high cholesterol level in the blood is considered one of the major predisposing factors for the development of atherosclerotic plaques in the arteries. *Lactobacillus* in the intestine plays an important role in assimilating cholesterol and the metabolism of triglycerides and fatty acids. The proposed mechanism of action includes: 1) Bile salts degradation through the action of microbial bile salt hydrolase (BSH), making them relatively less soluble and thus reducing their reabsorption by the intestinal epithelial layer and increasing their excretion in feces as observed in *L. gasseri* LBM220 (Rastogi et al., 2021) and *L. mucosae* SRV5 and SRV10 (Rastogi et al., 2020) 2) Microbial conversion of cholesterol to relatively soluble form coprostanol which can readily assimilate and excreted out *via* feces 3) Incorporation of cholesterol by the microbial membrane as observed in strain *L. acidophilus* ATCC 43121 4) production of short chain fatty acids (SCFA). SCFA can directly impede hepatic cholesterol synthesis by inhibiting 3-hydroxymethyl-3-glutaryl-CoA reductase enzyme in the liver (Amiri et al., 2021). GM analysis revealed that *lactobacillus* supplementation in mice model, selectively promotes the SCFAs metabolizing commensals, such as Ruminococcus, Eubacterium and Roseburia, thereby facilitating higher levels of fermented SCFAs (Amiri et al., 2021). The study also showed *L. fermentum* 296 when given to high-fed rat model for 4 weeks, significantly increased HDL and reduced harmful LDL owing to their increased production of SCFA (Cavalcante et al., 2019). Further, certain *lactobacillus* strains are directly involved in hypercholesterolemia, such as *L. plantarum* 06CC2 which remarkably reduced serum low-density lipoprotein (LDL), triglycerides (TC), free fatty acid levels, apolipoprotein B and increase apolipoprotein A-I levels in HFD Balb/c mice. At the same time, this strain also targets hepatic tissues for lipid deposition by regulating the expression of enzymes involved in cholesterol metabolism (Yamasaki et al., 2020).

Apart from BAs and SCFAs, increasing evidence demonstrates that gut-derived microbial metabolites, particularly TMAO and LPS are also involved in the progression of CVD. Trimethylamine (TMA) is a metabolic product that is metabolized by gut commensals from dietary choline and l-carnitine which when absorbed reaches the liver where it is oxidized to TMAO. The high TMAO level causes inhibition of cholesterol reverse transport, pro-inflammatory changes in arterial vessel walls, induction of high cholesterol accumulation in macrophages, platelet hyper-responsiveness and arterial thrombosis (Zhao et al., 2021). Studies have shown *Lactobacillus* species alleviate TMAO-associated CVD risk by restraining the growth of gut microbes that produce key enzymes involved in TMA production. This was evidenced by research work carried out by Qiu et al., 2018, wherein *L. plantarum* ZDY04 intervention leads to significantly lowered TMAO circulating levels and TMAO-induced atherosclerosis by altering the relative microflora population of the genus Mucispirillum and the families Erysipelotrichaceae, Lachnospiraceae, and Bacteroidaceae in the animal model. *L. plantarum* Dad-13 supplementation in obese adults leads to a higher population of Bacteroidetes and lower Firmicutes flora in the gut (Rahayu et al., 2021). Also, *L. plantarum* supplementation directly reduced serum TMAO levels as well as circulating inflammatory factors of IL8, IL-12, and leptin in CVD patients. Furthermore, LPS as a bacterial outer membrane component also induces CVD by activating macrophages to secrete pro-atherosclerotic inflammatory cytokines (IL-1, TNF-α, IL-12, IL-6, IL-8) that accelerated atherosclerosis and heart failure occurrence owing to down-regulation of mitochondrial fatty acids oxidation in cardiomyocytes (Zhao et al., 2021). Also, LPS increases platelet aggregation through TLR4-mediated leukocyte cathepsin G activation causing thrombosis (Zhao et al., 2021). The *L. brevis* alleviated CVD onset by blocking LPS-induced inflammatory cytokine expression in the host (Chang et al., 2016). Similarly, *L. paracasei* FZU103 was found to modulate the LPS-induced inflammatory axis and cholesterol metabolism in mice fed with HFD. Metagenomic studies also demonstrated shift in GM, with higher abundance of *Alistipes*, *Ruminococcus*, *Helicobacter* and *Pseudoflavonifractor* but lower population of *Tannerella*, *Blautia*, and *Staphylococcos* in gut (Lv et al., 2021). In *L. plantarum* LP91-fed LPS-induced mice, expression of atherosclerotic inflammatory factors, particularly TNF-α and IL-6, vascular cell adhesion molecule, E-selectin was found to be downregulated, thus showing improvement in CVD symptoms (Aparna Sudhakaran et al., 2013).

### 3.6 Metabolic disorders

Many recent publications have reported the beneficial role of *Lactobacillus* spp. in assuaging the onset of metabolic disturbances, such as obesity and diabetes mellitus, which are characterized by a low-grade inflammation (Archer et al., 2021).

#### 3.6.1 Obesity

Obesity is a multifaceted metabolic disease associated with changes in adipose tissues (AT), which is a complex endocrine organ involved in energy homeostasis. Usually, AT are subsuming adipocytes, lymphocytes, macrophages, fibroblasts, endothelial cells that produce plasminogen activator inhibitor (PAI-1), adiponectin, leptin, vascular regulators angiotensin II and cytokines (Andersen et al., 2016). The onset of obesity leads to dysfunctional AT with physiological changes in vascularization, oxidative stress levels, secreted adipokines and inflammatory state of infiltrated lymphocytes (Jo et al., 2009). In obese patients, a rise in free fatty acids (FFAs) level causes activation of signalling pathways, particularly TLRs which contribute to the pro-inflammatory response by increasing the production of molecules such as TNF-α, IL-6, IL1β, leptin, resistin, and chemokines in cells from lamina propria of gut (Khan et al., 2021). In the gut, activation of TNF-α stimulates apoptosis signalling pathways, FFAs levels and downregulates the expression of GLUT-4 transporters. In the high-fed diet (HFD) mice group, infiltration of CD8^+^ T cells, TNF-α, IFN-ɣ, and CX3CR1^int^ macrophages in adipose tissues in response to high glucose, FFAs and apoptosis leads to inflammation. Of note, one of the driver for inflammatory alterations and loss of gut epithelial integrity in obesity includes, leakage of bacterial endotoxin LPS (Khan et al., 2021). This leads to an inadequate distribution of lymphocytes, changes in cytokines levels, gut microbiota and immune response to dietary antigens. Much research has been centred on the importance of *lactobacillus* in treating obesity by alleviating lipid accumulation, oxidative damage, inflammation, and gut dysbiosis. A recent publication reported synergistic effects of *L. curvatus* HY7601 and *L. plantarum* KY1032 in lowering excess weight and fat accumulation in the HFD mice group with reduced inflammatory biomarkers (Park et al., 2013). Choi et al., 2020, reported weight loss in HFD-fed mice when treated with *L. plantarum* LMT1-48 with fewer lipids accumulation, immune cells infiltration in AT and adipocyte size. Notably, multiple strains of lactobacillus-particularly, *L. casei* IMVB-7280, *L. paracasei* HII0, *L. paracasei* CNCM I-4034, *L. rhamnosus* CGMCC1.3, *L. rhamnosus* LA68 and *L. casei* IBS041 have proved positive effects in reducing obesity symptoms, likely, reduced weight gain, lower cholesterol levels, lower adiposity and inflammation (Wiciński et al., 2020). Similarly, *L. reuteri* GMNL-263 ameliorated symptoms pertaining to obesity in HFD-fed rats by decreasing serum pro-inflammatory factors levels and remodeling white adipose tissue (WAT) energy metabolism (Chen et al., 2018). Besides, Joung et al., 2021 reported lower fat accumulation in AT in HFD-fed mice on *L. plantarum* administration, which was attributed to altered gut microbiota with reduced Firmicutes/Bacteroidetes ratio. Overall, *Lactobacillus* strains have achieved remarkable results in the treatment of obesity-related symptoms.

#### 3.6.2 Diabetes mellitus

Diabetes mellitus, a chronic metabolic disease is characterized by a consistently high serum glycemic index. A study of the Global Burden of Disease 2015 has reported that diabetes is one of the major causes of mortality in urban populations. There has been a significant association between the inflammatory condition and metabolic disturbances among diabetic patients. The postulated mechanism includes raised levels of the pro-inflammatory cytokine, TNF-α which inactivates insulin receptor (IRS-I) by phosphorylating serine residue. While other cytokines IFN-γ, TNF-α, and IL-1β works in a synergistic manner by infiltrating the β-cells of the pancreas, subsequently inducing cellular apoptosis and damage. Thus, higher pro-inflammatory cytokines levels in muscle, liver and adipose tissues are major drivers of diabetes pathology as they inhibit insulin signalling causing insulin resistance (Tsalamandris et al., 2019; Bezirtzoglou et al., 2021). Interestingly, *L. plantarum* Y44 showed downregulation of pro-inflammatory cytokine genes in the liver, intestine and muscle tissues particularly by activating regulatory anti-inflammatory cytokine IL-10, highlighting their immunomodulatory role (Liu et al., 2020). Likewise, *L. casei* Shirota strain showed reduced pro-inflammatory cytokines IL-4, IL-6 and C-reactive protein (CRP) levels in Streptozotocin (STZ)-induced diabetic rat models (Zarfeshani et al., 2011). Archer et al. (2021) also reported *L. fermentum* MCC2759 exhibited a reduction in glucose profile and pro-inflammatory cytokines, IL-10 in the liver, MAT, muscle and intestinal tissues in STZ-induced diabetic rats. This study also proved enhanced insulin sensitivity (GLP-1, GLUT-4, adiponectin), intestinal barrier integrity (ZO-1) and TLR-4 receptor expression. In addition to this, *L. casei* is reported to reduce serum glucose contents owing to improvement in post-immune responses *via* suppression of IL-2 and IFN- γ production (Qu et al., 2018). Another study by Park et al. (2015) reported that the anti-hyperglycemic effect on male db/db mice on administration of *L. rhamnosus* GG (LGG) is associated with increased ER stress and suppressed macrophages, leading to increased insulin sensitivity. Also, alteration in the expression of some diabetes-associated genes plays a role. Probiotic *L. rhamnosus* NCDC17 has reported to have antidiabetic capacity owing to its ability to upregulate mRNA expression of glucose metabolism and insulin sensitivity related genes such as GLUT4 (glucose uptake related genes), pp-1 (glycogen synthesis related genes) and PPAR- γ (insulin sensitivity related genes) and downregulating G6PC (gluconeogenesis related genes) (Singh et al., 2017). Further, consumption of probiotics affects the gut microflora composition which in turn alleviates intestinal epithelium and suppresses the immune response by reducing the TLR4 signalling pathway, ultimately increasing insulin sensitivity (Li et al., 2021). Some other postulated mechanisms include enhanced glucagon-like peptide-1 (GLP-1) secretion from intestinal L-cells to inhibit postprandial hyperglycemia by elevating the level of insulin released from pancreatic beta cells and reducing glucotoxicity (Kesika et al., 2019). A study involving *L. kafiranofaciens* M and *L. kefiri* K administration was found to stimulate GLP-1 secretion with concomitant rise in glucose metabolism (Kocsis et al., 2020).

### 3.7 Bone health (gut-bone axis)

Metabolic bone disorder, osteoporosis is characterized by poor bone tissues, deteriorated bone mass and porosity which subsequently lead to weak and brittle bones that are susceptible to fractures (Akkawi and Zmerly, 2018). Notably, *Lactobacillus* spp. as whole bacteria or their metabolites and/or structural components (such as SCFAs, hydrogen sulfide (H_2_S), insulin-like growth factor- I (IGF-I), LPS and peptidoglycans, etc) act as powerful immune cells controller and exhibit regulatory role in bone resorption and formation (Zaiss et al., 2019). Thereby, confirming their role as a linker in the “Gut-Bone axis”. Factors namely, IGF-I produced predominately in hepatic cells in response to dietary intake and gut microbes directly mediate the gut-bone axis (Zaiss et al., 2019). The microbial metabolite, H_2_S acts as a gasotransmitter, is produced by gut-resident Lactobacilli spp. and stimulates bone formation and postnatal skeletal development by activating Wnt signaling (Grassi et al., 2016). In osteoblasts, wnt signaling activation mediates enhanced osteoblastogenesis and arrests osteoblast apoptosis. Moreover, SCFAs have gained tremendous attention for their capacity to diffuse bone tissues and regulate immune responses (Zaiss et al., 2019). SCFA, particularly butyrate and propionate induce the proliferation of mature Treg cells. The maturation of Tregs depends upon GPR109a and GPR43 receptors expressed on dendritic cells (DC). The Treg cells suppress the CD4^+^ T cells that are present on the endosteal surfaces of the bone by producing immunosuppressive cytokines IL-10 and TGF-β; resulting in improved osteoporosis and bone mass density (BMD) by directly increasing osteoblast differentiation and reducing osteoclastogenesis (Smith et al., 2013). Also, activated Tregs cells are required for calciotropic parathyroid hormone–stimulated (PTH-stimulated) bone formation as it induces bone anabolism *via* Treg/Wnt10b/Wnt signaling pathway (Yu et al., 2018). Dietary supplementation with *L. rhamnosus* LGG for 4 weeks directly increased circulating and intestinal butyrate levels, confirming its capacity to diffuse from the intestine to distant organs like bone. In bone tissues, the butyrate induces Tregs and improves bone health. This study by Tyagi et al., 2018 further proved that *L. rhamnosus* LGG also altered gut microbial composition, with a higher Clostridia population that is known to elicit the generation of SCFAs in the gut. Thus, *L. rhamnosus* LGG substantially mediates the pathway linking SCFAs, Tregs, and bone formation.

To date, multiple studies have utilized animal models to determine *lactobacillus* efficacy in reducing both primary and secondary osteoporotic bone loss. Oral intake of *L. reuteri* ATCC 6475 by healthy male mice for 1 month resulted in improved bone mineral content, vertebral and femoral trabecular bone density, trabecular thickness and trabecular number when compared to untreated controls. Increased bone density leads to higher levels of bone formation rate as evidenced by the osteoblast marker osteocalcin. *L. reuteri* ATCC 6475 acts by systemically suppressing gene expression of pro-osteoclastogenic and pro-inflammatory cytokines in both the bone marrow and the intestine (Nilsson et al., 2018). These anti-inflammatory effects, which are also observed in other species of *Lactobacillus* tend to directly increase the calcium transport across the intestinal barrier. Immune cell activation largely depends on calcium levels. In case of hypocalcemia, treatment of rats with yogurt-enriched with *L. casei*, *L. reuteri* and *L. gasseri* improved calcium absorption in a PTH-dependent manner. Likewise, *L. rhamnosus* (HN001) also improved calcium and magnesium retention in rat models (Collins et al., 2017). Ohlsson et al., 2014 treated mice with either a single *L. paracasei* strain (DSM13434) or a mixture of three strains (*L. plantarum* DSM 15312, DSM 1531 and *L. paracasei* DSM13434) in the water for 14 days resulted in increased cortical bone mineral content and decreased levels of urinary fractional excretion of calcium and resorption marker C-terminal telopeptides as compared to control. Different *Lactobacillus* strains act *via* distinct and/or overlapping pathways, such as *L. helveticus* was found to not only increase bone density by elevating calcium uptake, but also secrete bioactive peptides valyl-prolyl-proline (VPP) and isoleucyl-prolyl-proline (IPP) (Parvaneh et al., 2018). Further, conversion of insoluble inorganic salts into soluble forms, protection of intestinal mineral absorption sites, triggering of the modulation of calcium-binding proteins, and minimization of the interaction of minerals with phytic acids are the main actions reported by different strains of *Lactobacillus* that supported bone health.

## 4 Future prospects

An exponential advancement in sequencing processing, genome assembly and annotation technologies, has resulted in thousands of publicly available genomes of *Lactobacillus* spp. Access to these data has revolutionized the molecular view of probiotic bacteria, significantly accelerating the research deciphering the complexity associated with interactions between resident microbiota and the mucosal immune system. Notably, advancements in genomic tools, particularly functional genomics, proteomics, transcriptomics, and secretomics have helped in understanding the intricate dynamic host-microbe crosstalk that extends far beyond gastrointestinal health. To exemplify, studies on the bi-directional crosstalk between the GIT and the brain (gut-brain axis) are revealing the neurochemical importance of gut homeostasis. Similarly, studies involving the GIT and the lungs crosstalk (gut-lung axis) highlight the significance of microbial metabolites in regulating host’s immune responses. In addition this, *Lactobacillus* spp. modulate mucosal immunity through the interaction of proteinacious microorganism-associated molecular patterns (MAMPs) with pattern recognition receptors (PRRs) on antigen-presenting cells (APCs), such as dendritic cells and macrophages. Recently, the proteomic and genomic profiles of several lactobacilli were bioinformatically screened to create a secretome database cataloging the various extracellular proteins. For example, a proteomic-based method was used to identify S-layer associated proteins (SLAPs) in *L. acidophilus*. After extraction, the SLAPs were identified through mass spectrometry and referenced to the secretome database. The mutational analysis of SLAPs showed immunomodulatory phenotype using *in vitro* bacterial-DC co-incubation assays (Huang et al., 2021). However, it is important to note that the transcriptional networks induced by each probiotic were unique to each strain studied and that they show distinct metabolic and immunogenic profiles in the host. Thus, there is a growing need for human clinical trials with experimental designs, reflecting the future progress that has been made in the field of probiotics and GIT microbiome research.

## 5 Conclusion

Taken together, the intestinal microbial species, particularly Lactobacilli spp., within gut are identified as potential and talented players in restoring gastrointestinal barrier function, immune stimulation and gut microbial flora. So far, we have highlighted the intricate crosstalk between gut-dwelling Lactobacilli spp., or their metabolites and the immune components in distant organs to promote host’s health. The healthy balance in the intestinal ecosystem is preserved by the circuitry of monitoring mechanisms between potentially pro-inflammatory cell [Th cells secreting IFN-y, Th17 cells that secrete interleukin IL-17, and IL-22], and anti-inflammatory Foxp3+ receptor Tcells. Certain strains of lactobacilli stimulate the anti-inflammatory fork of the adaptive immune system by controlling Treg maturation or by driving IL-10 and IL-12 production. Given the current epidemic of inflammatory disorders plaguing present society, a call is necessary for feasible, available, and safe treatments to prevent and fight against it. Even though inflammatory and metabolic disorders pathogenesis is multifactorial and highly complexed, yet recent literature suggests modulation of gut microbial flora and immune responses using probiotics as the primary therapeutic intervention. Consequently, gut microbiome modulation to preserve a stable, consistent metabolic environment may be helpful in preventing and as additional treatment in affected patients. In future, far more in-depth clinical studies will be required to substantiate the therapeutic approaches with *lactobacillus* in directly maintaining gut microbiota homeostasis and regulate functional metabolites (such as TMAO, SCFAs, BAs, and LPS) which further lower risks associated with immune inflammation, high lipid cholesterol and oxidative stress. From author’s viewpoint, those looking to ameliorate their overall health by improving their gastrointestinal microbial complexity might find it more beneficial to target on consuming a fermented diet (such as yoghurt, kimchi, miso, sauerkraut) rich in *Lactobacillus* spp. To conclude, therapy with *Lactobacillus* spp. still provides a potential frontier in the treatment and prevention of inflammatory diseases.
